# The effect of *Opuntia ficus‐indica* juice supplementation on oxidative stress, cardiovascular parameters, and biochemical markers following yo‐yo Intermittent recovery test

**DOI:** 10.1002/fsn3.529

**Published:** 2017-12-06

**Authors:** Aloui Khouloud, Salma Abedelmalek, Hamdi Chtourou, Nizar Souissi

**Affiliations:** ^1^ Research Laboratory ‘‘Sports performance optimization’’ National Center of Medicine and Science in Sports (CNMSS) Tunis Tunisia; ^2^ Faculty of Sciences of Bizerte University of Carthage Bizerte Tunisia; ^3^ Department of Physiology and functional explorations Sousse Faculty of Medicine Sousse Tunisia

**Keywords:** biochemical response, cardiovascular response, exercise, oxidative stress, Supplementation

## Abstract

The aim of the study was to investigate the effect of a flavonoid‐rich fresh fruit juice on cardiovascular, oxidative stress, and biochemical parameters during the yo‐yo intermittent recovery test (YYIRT). Twenty‐two healthy males subjects participated in this study divided into two groups: An experimental group (EG:* n* = 11) who consumed the antioxidant supplement and a control group (CG:* n* = 11). All participants performed two test sessions at 07:00 hr before and after 2 weeks of supplementation with *Opuntia ficus‐indica* juice. Blood samples were taken before (P1) and immediately (P2) after the YYIRT. Our results showed that following the 2,2‐diphenyl‐1‐ picrylhydrazyl (DPPH ^●^) test, the *Opuntia ficus‐indica* juice has an antioxidant capacity for capturing free radicals (*p* < .05) and reducing oxidative stress related to exercise. Concerning biochemical and cardiovascular parameters, our results showed a significant increase on total cholesterol (TC) (*p* < .01), triglycerides (TG) (*p* < .05), high‐density lipoprotein (HDL) (*p* < .01), low‐density lipoprotein (LDL) (*p* < .01), creatine kinase (CK) (*p* < .01), lactate deshydrogenase (LDH) (*p* < .01), glucose (GLC) (*p* < .01), systolic (SBP), and diastolic blood pressure (DBP) (*p* < .01) immediately after exercise. However, TC (*p* < .05), TG (*p* < .05) and LDL (*p* < .05), the maximal heart rate (HRmax), the CK (*p* < .05), and LDH (*p* < .01) as well as the malondialdehyde (MDA) (*p* < .01) demonstrated a significant decrease after supplementation of *Opuntia ficus‐indica* juice before and immediately after YYIRT. However, no significant effect on HDL (*p* > .05), GLC (*p* > .05) levels nor the SBP and DBP (*p* > .05) was observed after supplementation with *Opuntia ficus‐indica* juice. The supplementation leads to an improvement on YYIRT performance (The total distance covered during the YYIRT,*V*O_2max_, VMA) and the rating of perceived exertion (RPE). *Opuntia ficus‐indica* juice has a potent antioxidant activity that reduces total and LDL‐cholesterol with only a moderate lowering of HDL‐cholesterol and oxidative stress. Moreover, supplementation decreases muscle damage caused by the endurance exercise.

## 1 INTRODUCTION

It has been established that increased consumption of flavonoids, present in the majority of fruits and vegetables, has beneficial health effects (Hooper et al., 2008). In fact, it is related to decreased risk of cardiovascular disease and cancer (Hooper et al., 2008; Kasote, Katyare, Hegde, & Bae, 2015). Among these fruits include the *Opuntia ficus‐indica* which is already used for treating numerous diseases such (i.e., ulcers, allergy, rheumatism, obesity) (Feugang, Konarski, Zou, Stintzing, & Zou, 2006; Padilla‐Camberos et al., 2015). Indeed, these fruits, beyond the nutritional benefits, its consumption are beneficial to human health due to its antioxidant functions (Tesoriere, Fazzari, Angileri, Gentile, & Livrea, 2008). Moreover, *Opuntia ficus‐indica* contains compounds with anti‐radical activity (i.e. phenolics, flavonoids, and pigment compounds) (Maataoui, Hmyene, & Hilali, 2006). These compounds were found to have a high potential for capturing free radicals. Maataoui et al. (2006) investigated the antioxidant activity of *Opuntia ficus‐indica* juice, by the DPPH^●^ test. The juice from purple color has higher antioxidant activity that those from fruits of yellow‐orange markers of stress color (Maataoui et al., 2006). Strenuous exercise was reported to increase the generation of reactive oxygen species (ROS) by cells, which may lead to cellular damage (Jówko, Długołęcka, Makaruk, & Cieśliński, 2015). These ROS are considered as a potent oxidation stress indicator in biological systems (El Abed et al., 2011; Wang, Wu, Wu, & Wei, 2013). Hammouda, Chaouachi, Ferchichi, Kallel, & Souissi (2012)) proved a significant increase in blood lactate and an increase in biochemical markers of muscle damage (i.e., creatine kinase (CK) and LDH) and lipid profile during YYIRT. Although, research suggests that antioxidant supplementation has a potent role in postexercise recovery in athletes (Jówko et al., 2015). Hence, elite athletes are more vulnerable, than a sedentary person, to physical performance altered and physiological responses due to stress caused by free radical production (Hammouda et al., 2012; Prahalathan, Saravanakumar, & Raja, 2012). Several studies revealed that antioxidant supplements led a great improvement of mental/physical performance (Nikolaidis, Kerksick, Lamprecht, & McAnulty, 2012; Strobel et al., 2011).

Overall, the best performance is generally observed in the afternoon. Thus, studies in the domain continue to find strategies to counteract this decrease of performance in the morning.

Thus, 2‐week or 4‐week of antioxidant supplementation is recommended after acute exercise in order to delay muscle recovery (Jówko et al., 2015; Knab et al., 2013). However, these findings concerning the training adaptation after antioxidant supplementation was not confirmed by other studies (Higashida, Kim, Higuchi, Holloszy, & Han, 2011; Yfanti et al., 2010). In this context, the supplement of antioxidants pre‐exercise has been recently a topic of current debate and maybe should be re‐evaluated (Knab et al., 2013). These studies did not take into consideration the impact of natural antioxidant fruit supplementation, especially on *Opuntia ficus‐indica* juice.

But one study mentioned in the literature in vivo. It is of Deldicque et al. (2013) who demonstrated that chronic *Opuntia ficus‐indica* supplementation (i.e., specific extract fruit: capsule) could be an important nutritional strategy for muscle glycogen resynthesis post‐exercise in healthy subjects. No study found in the literature that looks into the impact of an *Opuntia ficus‐indica* supplementation on cardiovascular parameters and bio markers of oxidative stress after physical exercise. It is critical therefore to investigate the outcome of 2 weeks supplementation with *Opuntia ficus‐indica* juice on cardiovascular parameters oxidative stress, and biochemical markers of muscle damage following endurance exercise. The aim of this study was to evaluate the effect of 2‐week *Opuntia ficus‐indica* juice supplementation on biochemical parameters, oxidative stress markers, muscle damage, and glucose (GLC) in healthy subjects. We hypothesized that *Opuntia ficus‐indica* juice has an antioxidant action and its supplementation improves performance following the YYIRT with the modification of the antioxidant profile.

## 2 MATERIAL AND METHODS

### 2.1 Participants

Twenty two healthy male athletes (Table 1) volunteered for the study participated in this study. In randomized order, participants were divided into two groups; an experimental group (EG: *n* = 11) and control group (CG: *n* = 11). After receiving a detailed explanation of the protocol, they gave written consent to participate in this study. This study protocol was in agreement with the Helsinki Declaration for human experimentation and was approved by the university ethics committee. The participants were also selected based on their chronotype and on the basis of their answers to self‐assessment questionnaire of Horne & Ostberg (1975). They had an intermediate chronotype (i.e., sleep duration between 23:00 ± 1:00 and 07:00 ± 1:00 hr) and kept standard times for eating prior to the beginning of the study (Abedelmalek, Chtourou, Souissi, & Tabka, 2015).

**Table 1:** (Mean ± SE) values for daily nutrition consumption and anthropometric parameters in both group

	CG (*n* = 11)	EG (*n* = 11)	*p* value
Age (years)	20.91 ± 1.22	21.00 ± 0.84	NS; *p* = .88
Height (cm)	181.82 ± 8.13	181.36 ± 2.28	NS; *p* = .89
Weight (kg)	73.10 ± 1.90	72.75 ± 1.79	NS; *p* = .89
BMI (kg/m2)	22.13 ± 0.49	22.15 ± 0.54	NS; *p* = .97
Energy intake (Kcal/day)	3084.9 ± 74.1	2905.3 ± 69	NS; *p* = .82
Protein (g/day)	107.7 ± 4.9	104.3 ± 4.8	NS; *p* = .93
Fat (g/day)	89.5 ± 1.9	84.6 ± 2.1	NS; *p* = . 73
Carbohydrate (g/day)	451.3 ± 12.07	420.1 ± 9.7	NS; *p* = .53
Vitamin E (g/day)	12 ± 0.3	12.2 ± 0.4	NS; *p* = .67
Vitamin C (g/day)	52.9 ± 0.2	51.3 ± 0.9	NS; *p* = .001

The exclusion criteria of this study were the use of tobacco products, alcohol consumption, sleep disorder, and use of any dietary supplements in 2 weeks prior to the study. Additionally, subjects suffering from diabetes mellitus, obesity, cardiovascular, pulmonary or renal disorders, or taking any antioxidant supplements were also excluded from the study.

### 2.2 Experimental design

**Figure 1:**
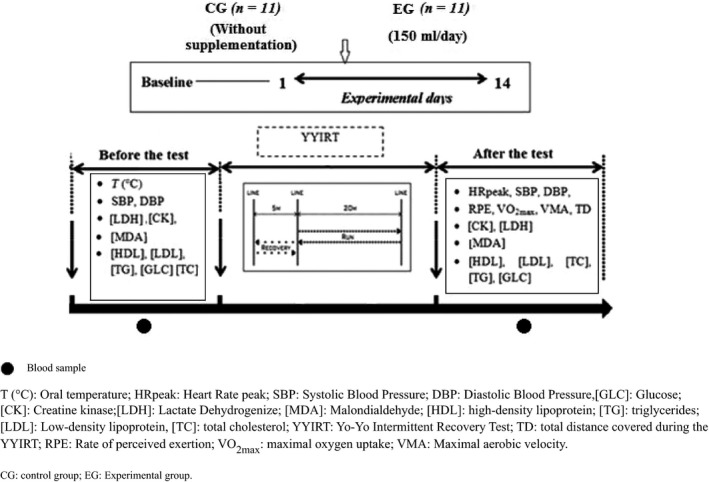
Experimental protocol

The experimental procedure is illustrated in Figure 1. A randomized, controlled, clinical study was conducted. During 2 weeks of treatment periods, *Opuntia ficus‐indica juice* supplementation of 150 ml/daily was utilized.

In this work, we are committed to checking the effect of *Opuntia ficus‐indica* supplementation on the antioxidant profile following the YYIRT. For the antioxidant power, 2‐2 diphenyl‐1‐picrylhydrazyl DPPH^●^ test was used. In order to minimize learning effects, participants came to the laboratory to be familiarized with the procedure during the week before the experiment. Before the test sessions, only one glass (150 to 200 ml) of water was allowed. The daily energy intake goal was (~3,000 kcal) per capita/day. In a randomized order, subjects participated in two experimental sessions. The first was a baseline condition in which subjects were taking a normal diet (without *Opuntia ficus‐indica* juice supplementation). The second is a condition of *Opuntia ficus‐indica* juice supplementation for 2 weeks (Tesoriere, Butera, Pintaudi, Allegra, & Livrea, 2004). The experimental group (EG) takes supplementation of 50 ml of *Opuntia ficus‐indica* juice 3 times daily (Pilaczynska‐Szczesniak, Skarpanska‐Steinborn, Deskur, Basta, & Horoszkiewicz‐Hassan, 2005). The juice was prepared fresh every day. A control group (CG) does not take any antioxidant throughout the experimental period. Before and after 2‐weeks treatment period, subjects performed the yo‐yo intermittent recovery test (YYIRT Level 1) at the same time of day (07:00–09:00 hr) in the same order. The test was performed, at air temperature between 27 and 28°C and in 53%–56% relative humidity.

At the beginning of each session, the weight and body composition of subjects were recorded using bioelectrical impedance scale (Tanita, Tokyo, Japan) to the nearest 0.1 kg calibrated by one trained technician. The heart rate and blood pressure were monitored, using Automatic Blood Pressure (Microlife, W90, Paris). Blood samples were taken at rest and after YYIRT.

### 2.3 Dietary intake

The participants were asked to keep a similar diet for the treatment periods except refrain the use of any antioxidant products. Subjects filled out a 24‐hr dietary record during this period. The total dietary intake (i.e., vitamins, macro nutrient, and energy) was estimated with a dedicated picture book and calculated, using the NUTRISOFT BILNUT (Version 2.01 Paris, France) (based on national food tables) (Kunachowicz, Nadolna, Przygoda, & Iwanow, 2005).

### 2.4 Antiradical capacity by DPPH^●^

The tests, using 2, 2‐diphenyl‐1‐ picrylhydrazyl (DPPH^●^) radical are the most of the methods applied in the determination of antiradical activity. One of the reasons is that this method is simple and sensible. DPPH^●^ assays are based along the same rule as that described by Brand‐Williams, Cuvelier, & Berset (1995), but the analytic protocols differ in more parameters (i.e., absorbance, reaction time, the reference solution). DPPH^●^, a purple‐colored free radical, was decreased into the yellow‐colored diphenylpicryl hydrazine. The method used in our study on the *Opuntia ficus‐indica* juice is that described by Tuberoso, Kowalczyk, Sarritzu, & Cabras (2007). An ethanolic solution (2.5 mg/100 ml) of the stable DPPH^●^ radical was prepared and applied for the assay. *Opuntia ficus‐indica* juice is dissolved in 10 ml of ethanol and it was placed into test tubes. Then, it was added to samples at 250 μl of the ethanolic solution. The test tubes were incubated for 30 min, and the optical density was read at 520 NM against the ethanol in a UV spectrometer (Genesys‐10, Madison, USA). The radical scavenging activity was estimated utilizing the following recipe:

$$\%\;\;\text{scavenging activity}\;=\;(\left(A_{\mathrm{control}}\;-\;A_{\mathrm{sample}\;\mathrm{extract}}\right)/A_{\mathrm{control}})\;\times \;100$$

#### 2.4.1 Sample and treatments

*Opuntia ficus‐indica* fruit with purple color was selected from a local market in Tunisia in the springtime of 2013. Only fruit without external injuries were chosen, washed, and peeled manually. To extract the juice, the pulp was pressed using an industrial mixer (Kenwood, 700W, China) and was then drawn through a strainer to remove seed enough to get the maximum of Juice. During 2 weeks of treatment, *Opuntia ficus‐indica juice* supplementation, subjects were asked to receive of 150 ml/daily.

### 2.5 Exercise protocol

#### 2.5.1 The yo‐yo intermittent recovery test level‐1

According to the procedures suggested by Krustrup et al. (2003), the YYIRT was performed. It consisted of 20‐m shuttle runs performed at increasing velocities with 10 s of active recovery at a distance of 5‐m until exhaustion. The goal of the trial was seen when the participant twice failed to hit the front line in time or he felt unable to make out another shuttle. The total distance covered during the YYIRT was considered as the test score.

#### 2.5.2 Rate of perceived exertion (RPE)

Before performing exercise testing, the RPE (Borg, 1982) scales were explained to each participant. The scale presents a 15‐point scale ranging from 6 (very, very light) to 20 (very, very hard). Thereby, during exercise testing, overall RPE, and physical stress was assessed. RPE is considered as a method of measure of training in response to different modes of exercise (Impellizzeri, Rampinini, Coutts, Sassi, & Marcora, 2004).

### 2.6 Blood samples and analyses

Blood samples were taken in the antecubital vein, sitting, before (P1), and 3 min after (P2) completing the YYIRT during two experimental conditions (i.e., before supplementation and after supplementation) (Hammouda et al., 2012; Jówko et al., 2015). The test venous blood samples were drawn into heparinized and EDTA test tubes (4 ml), centrifuged (for 20 min at 3000 RPM) and frozen, and stored at ‐80 C until analysis of selected blood markers.

### 2.7 Biochemical markers

The concentrations of high‐density lipoprotein (HDL)‐cholesterol and LDL‐cholesterol were determined by a colorimetric enzymatic assay: direct test in homogeneous phase (liquicolor) (Kit; HUMAN, Ref: 10084: Gesellschaft für Biochemica und Diagnostica mbH Wiesbaden, Germany). Concerning the TC assay, it was determined by the enzymatic method (CHOD — PAP) (Biomagreb; CHOLESTEROL; GHOD‐PAP, France), using the same protocol with sample incubation at 37°C for 5 min. The concentrations of TG were determined by the enzymatic method (GPO— PAP) (Biomagreb; CHOLESTEROL; GPO‐PAP, France). With regard to the enzymatic GLC assay, it is carried out using a photo metric enzyme assay in the presence of the enzyme glucose oxidase and peroxidase (GOD — PAP) (Biomagreb; GLUCOSE; GOD‐PAP; France).

Concerning muscle damage markers, CK levels were made by the diagnostic reagent (CK‐NAC FS) CQN code: L1 on serum (DiaSys; Diagnostic System GmbH, Germany). The UV method (ultra‐violet) was applied. Regarding the LDH concentrations, they were determined by the kinetic method of the French Society of Clinical Biology (SFBC) (Biomaghreb; France).

### 2.8 MDA‐TBARS s

Lipid peroxidation was assayed by determining the production of TBARS Thiobarbituric acid‐reactive substances (TBARS) Malondialdehyde (MDA). The measurement of malondialdehyde (MDA) was determined, using a spectrophotometric method based on the reaction between MDA and thiobarbutiric acid (TBA). First, 200 μl of plasma was added to 200 μl of 30% (w/v) trichloroacetic acid (TCA). After that, the blend was centrifuged at 3500 rpm for 10 min. To 800 μl of 0.4% (w/v) TBA was added 200 μl of the supernatant and all mix was incubated in a shaking water bath at 90°C for 10 min and then put directly into ice water. The concentration of MDA (determined at 532 NM) was expressed in micro mole per liter of plasma.

### 2.9 Statistical analysis

Statistical tests were processed using STATISTICA Software (StatSoft, France). Data were reported as mean ± ES. Once the normality was confirmed, using the Shapiro–Wilk‐W‐test parametric tests were performed. YYIRT parameter (i.e., *V*O_2max_, distance, HR and the RPE scores) were analyzed using a two‐way ANOVA with repeated measures (2 (group) × 2 (conditions)). Biochemical parameters (i.e., total cholesterol, triglycerides, HDL‐cholesterol, LDL‐cholesterol, LDH, CK, GLC), blood pressure and MDA data were studied utilizing a three‐way ANOVA with repeated measures (2 (group) × 2 (conditions) × 2 (points of measurement)). The Bonferroni post hoc test was performed whenever significant effects or a significant interaction was found using ANOVA. Anthropometric parameters were examined by a paired Student's t‐test. To assess the data practical significance, effect Size was calculated as partial eta‐squared, (η_p_^2^). The level of statistical significance was set at *p* < .05.

## 3 RESULTS

### 3.1 The anti‐radical activity of *Opuntia ficus‐indica* juice

**Figure 2:**
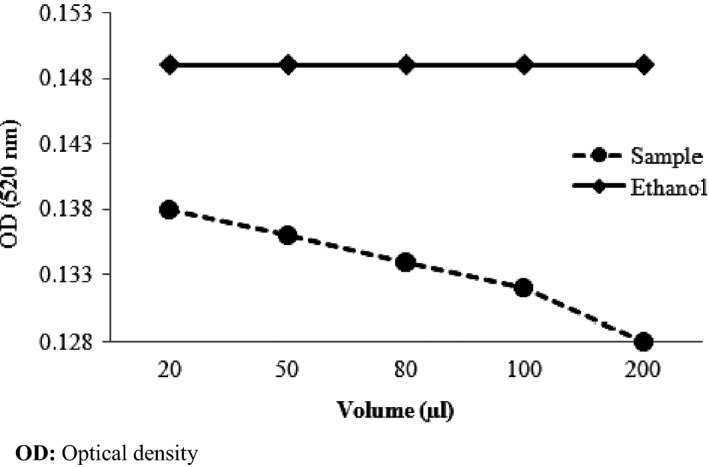
Antioxidant effect depending on the volume of the extract of *Opuntia ficusindica* and ethanol

The *t* student test for independent samples showed a significant dose‐dependent decrease (*t* = −8.95; *p* < .05) for the *Opuntia ficus‐indica* juice extracts compared with ethanol solution. In fact, all the extracts inhibited DPPH^●^ with a minimum 10.3% during 30 min of incubation. Thus, the *Opuntia ficus‐indica* introduced an anti‐radical activity significantly higher compared to the ethanol solution (Figure 2).

### 3.2 The YYIRT performances

The analysis of variance ANOVA revealed a significant group effect (*F* = 6.36; $\eta_{p}^{2}$ = 0.54; *p* < .05) and a significant condition effect (*F* = 6.59; $\eta_{p}^{2}$ = 0.59; *p* < .05) for the total distance traversed during the YYIRT and, *V*O_2max_ and VMA estimated. However, no significant interaction (*F* = 2.83; $\eta_{p}^{2}$ = 0.04; *p* > .05) was observed between these two factors (group × conditions). The post hoc analysis demonstrated that the *Opuntia ficus‐indica* juice supplementation leads to an improvement for the *V*O_2max_, VMA and the total distance covered during the YYIRT for EG (*p* < .05) (Table 2).

**Table 2:** Values (mean ± SE) of YYIRT parameters and RPE before and after supplementation

	EG		CG	
	Before supplementation	After supplementation	Before supplementation	After supplementation
*V*O_2max_ (ml/min/kg)	48.07 ± 0.57	49.67 ± 0.54^a^, ^b^	47.42 ± 0.62	47.73 ± 0.76
VMA (km.h^−1^)	14.11 ± 0.26	14.70 ± 0.20^a^, ^b^	13.87 ± 0.23	13.99 ± 0.25
Distance (m)	978.18 ± 97.74	1200.00 ± 74.543^a^, ^b^	887.27 ± 85.59	930.91 ± 93.74
RPE	16.18 ± 0.52	14.55 ± 0.44^a^, ^b^	16.82 ± 0.30	16.18 ± 0.23

VMA, Maximal aerobic speed; RPE, Rate of perceived exertion; EG, Experimental group; CG, Control group; YYIRT, Yo‐Yo intermittent recovery test.

a Significant difference compared with before supplementation (*p* < .05).

b Significant difference after supplementation for EG versus CG (*p* < .05).

### 3.3 The RPE scores

Statistical analysis showed a significant group effect (*F* = 8.391, *p* < .05) and a condition effect (*F* = 15.92; *p* < .01) for the RPE scales. However, the interaction between these two factors (group × conditions) was not significant (*p* > .05).

The post hoc analysis revealed that the RPE scales registered following the YYIRT present a significant decrease after *Opuntia ficus‐indica* juice supplementation for EG.

### 3.4 The cardiovascular parameters

The cardiovascular parameters are presented in Table 3. The ANOVA revealed that the supplementation was associated with an increase in the HRmax (*F* = 9.92; $\eta_{p}^{2}$ = 0.65; *p* < .05) with no significant group effect (*F* = 3.36; $\eta_{p}^{2}$ = 0.04; *p* > .05). Likewise, there was a significant interaction (group × conditions) (*F* = 6.14; $\eta_{p}^{2}$ = 0.49; *p* < .05). Concerning point of measurement (before and after YYIRT), the analysis revealed a significant effect for DBP (*F* = 45.14; $\eta_{p}^{2}$ = 0.78; *p* < .01) and SBP (*F* = 114.31; $\eta_{p}^{2}$ = 0.52; *p* < .01). However, no significant effect neither group, nor supplementation and no significant interaction (group × conditions × points of measurement) was observed for SBP or DBP (*p* > .05). The post hoc analysis showed that DBP and SBP values registered present a significant increase after YYIRT compared with baseline for both EG and CG.

**Table 3:** Cardiovascular parameters (mean ± SE) measured Before (P1) and After (P2) the YYIRT, before and after supplementation

	EG				CG			
	Before supplementation		After supplementation		Before supplementation		After supplementation	
	P1	P2	P1	P2	P1	P2	P1	P2
DBP (mmHg)	81.91 ± 2.64	97.73 ± 2.53	82.00 ± 4.14	93.73 ± 2.50^b^	81.45 ± 1.55	92.73 ± 1.51	84.18 ± 1.96	91.91 ± 1.82^b^
SBP (mmHg)	133.82 ± 2.29	159.82 ± 2.54	131.09 ± 4.04	156.64 ± 1.61^b^	133.91 ± 2.11	157.45 ± 1.71	134.55 ± 1.75	157.27 ± 2.27^b^
HRmax (bpm)		192.81 ± 1 .33		188.90 ± 1.71^a^		193.81 ± 1.57		192.54 ± 1.50

DBP, diastolic blood pressure; SBP, systolic blood pressure; HRmax, maximal heart rate; EG, Experimental group; CG, Control group; YYIRT, Yo‐Yo intermittent recovery test.

a Significant difference compared with supplementation (*p* < .05).

b Significant difference following the YYIRT (*p* < .05).

### 3.5 MDA‐TBARS

Concerning MDA, no significant difference effect group was observed (*F* = 1. 55; $\eta_{p}^{2}$ = 0. 06; *p* > .05). However, the analysis of variance revealed a significant condition effect (*F* = 20.45; $\eta_{p}^{2}$ = 0.68; *p* < .01) and point of measurement effect in (*F* = 17.54; $\eta_{p}^{2}$ = 0.45; *p* < .01).

The Post hoc test showed that the MDA decrease after supplementation with *Opuntia ficus‐indica* juice for the EG (*p* < .01). Likewise, MDA levels increase after the YYIRT in EG (*p* < .01) (Table 4). Nevertheless, no significant alteration was observed concerning CG (*p* > .05).

**Table 4:** Biochemical markers and MDA (mean ± SE) measured Before (P1) and After (P2) the YYIRT, before and after supplementation

	EG				CG			
	Before supplementation		After supplementation		Before supplementation		After supplementation	
	P1	P2	P1	P2	P1	P2	P1	P2
LDL‐ Cholesterol (mmol.l^−1^)	0.96 ± 0.07	1.29 ± 0.05^a^	0.58 ± 0.03	1.02 ± 0.03^a^, ^b^	0.89 ± 0.04	1.1 ± 0.06^a^	0.88 ± 0.04	1.11 ± 0.05^a^
HDL‐ Cholesterol (mmol.l^−1^)	0.76 ± 0.04	1.18 ± 0.07^a^	0.77 ± 0.04	1.19 ± 0.07^a^	0.77 ± 0.03	1.03 ± 0.02^a^	0.89 ± 0.06	1.06 ± 0.06^a^
Total Cholesterol (mmol.l^−1^)	2.93 ± 0.16	3.83 ± 0.15^a^	2.53 ± 0.09	3.49 ± 0.17^a^, ^b^	2.52 ± 0.09	3.54 ± 0.13^a^	2.51 ± 0.08	3.61 ± 0.12^a^
Triglycerides (mmol.l^−1^)	0.89 ± 0.05	1.40 ± 0.12^a^	0.78 ± 0.07	1.03 ± 0.05^a^, ^b^	0.78 ± 0.07	1.10 ± 0.05^a^	0.82 ± 0.06	1.09 ± 0.05^a^
LDH (UI.l^−1^)	264.54 ± 24.27	420.9 ± 28.6^a^	232.36 ± 14.9	283.72 ± 16.58^a^, ^b^	269.9 ± 8.97	438.36 ± 25.2^a^	271.9 ± 8.82	449.9 ± 21.3^a^
CK (UI.l^−1^)	170.63 ± 16.01	268.18 ± 27.09^a^	121.7 ± 5.9	184.63 ± 19.45^a^, ^b^	165.8 ± 2.56	241.09 ± 9.31^a^	167.18 ± 2.27	242.27 ± 11.5^a^
GLC (mmol.l^−1^)	4.88 ± 0.10	5.97 ± 0.09^a^	4.63 ± 0.05	5.66 ± 0.13^a^	4.37 ± 0.13	5.62 ± 0.11^a^	4.38 ± 0.12	5.66 ± 0.11^a^
MDA (μmol/L)	0.93 ± 0.02	1.02 ± 0.02^a^	0.80 ± 0.02	0.87 ± 0.01^a^, ^b^	0.95 ± 0.03	1.02 ± 0 .05	0.9 ± 0.04	0.91 ± 0.03

LDL, low‐density lipoprotein; HDL, high density lipoprotein; LDH, lactate dehydrogenase; CK, creatine kinase; GLC, blood glucose; MDA, malondialdehyde; EG, Experimental group; CG, Control group; YYIRT, Yo‐Yo intermittent recovery test.

a Significant difference following the YYIRT (*p* < .05).

b Significant difference EG versus CG after supplementation (*p* < .05).

### 3.6 Markers of muscle damage

Our results showed that LDH (*F* = 86.79; $\eta_{p}^{2}$ = 0.62; *p* < .01), the CK (*F* = 73.68; $\eta_{p}^{2}$ = 0.85; *p* < .01) increase significantly after the YYIRT. Indeed, statistical analysis indicated a significant decrease for the LDH (*F* = 19.76; $\eta_{p}^{2}$ = 0.54; *p* < .01) and the CK (*F* = 9.23; $\eta_{p}^{2}$ = 0.75; *p* < .05) after supplementation with *Opuntia ficus‐indica* juice. Likewise, a no significant group effect for the CK (*F* = 2.05; $\eta_{p}^{2}$ = 0.04; *p* > .05) was observed. However, a significant group effect was observed for LDH (*F* = 10.96; $\eta_{p}^{2}$ = 0.49; *p* < .01). Likewise, the interaction (group × conditions × points of measurement) for plasma concentrations of LDH (*F* = 37.20; $\eta_{p}^{2}$ = 0.38; *p* < .01) was significant. Nevertheless, there was no significant interaction for the CK levels (*F* = 0. 96; $\eta_{p}^{2}$ = 0.01; *p* > .01).

### 3.7 The biochemical markers

The biochemical markers are presented in the Table 4. ANOVA analysis showed a significant point of measurement effect for the LDL‐cholesterol (*F* = 6.93; $\eta_{p}^{2}$ = 0.81; *p* < .05), the HDL‐cholesterol (*F* = 84. 24; $\eta_{p}^{2}$ = 0.87; *p* < .01), the TC (*F* = 73.92; $\eta_{p}^{2}$ = 0.69; *p* < .01), the TG (*F* = 16.51; $\eta_{p}^{2}$ = 0.73; *p* < .05) and the GLC (*F* = 160.49; $\eta_{p}^{2}$ = 0.83; *p* < .01) concentrations. Likewise, statistical analysis indicated a significant condition effect for only the LDL‐cholesterol (*F* = 6.84; $\eta_{p}^{2}$ = 0.70; *p* < .05), the TC (*F* = 5.22; $\eta_{p}^{2}$ =** **0.52; *p* < .05), the TG (*F* = 6.55; $\eta_{p}^{2}$ = 0.52; *p* < .05), and a no significant condition effect was observed for HDL‐cholesterol (*F* = 1.37; $\eta_{p}^{2}$ = 0.03; *p* > .05) and GLC (*F* = 3.19; $\eta_{p}^{2}$ = 0.05; *p* > .05). Similarly, a non significant group effect on HDL‐cholesterol *F* = 0.43; $\eta_{p}^{2}$ = 0.05; *p* > .05), TC (*F* = 3.43; $\eta_{p}^{2}$ = 0.03; *p* > .05) and TG (*F* = 3.31; $\eta_{p}^{2}$ = 0.05; *p* > .05) was revealed. However, a significant group effect was detected for LDL‐cholesterol (*F* = 65.52; $\eta_{p}^{2}$ = 0.76; *p* < .05) and GLC (*F* = 17.08; $\eta_{p}^{2}$ = 0.65; *p* < .01). Likewise, the interaction (group × conditions × points of measurement) for plasma concentrations of TC (*F* = 0.52; $\eta_{p}^{2}$ = 0.05; *p* > .01), TG (*F* = 2.26; $\eta_{p}^{2}$ = 0.03; *p* > .05), HDL‐cholesterol (*F* = 0. 57; $\eta_{p}^{2}$ = 0.03; *p* > .05), GLC and LDL‐cholesterol was not significant.

Post hoc analysis showed that exercise leads to a significant increase for the LDL‐cholesterol, the HDL‐cholesterol, the TC, the TG and the GLC concentrations after the YYIRT for EG and CG. Moreover, statistical analysis indicated a significant decrease for the LDL‐cholesterol, the TC and the TG after supplementation with *Opuntia ficus‐indica* juice for EG. While, HDL‐cholesterol and GLC didn't alters after supplementation.

## 4 DISCUSSION

Our results indicated that following the DPPH^●^ test the *Opuntia ficus‐indica* juice has an antioxidant capacity for catching free radicals. Moreover, concerning biochemical, oxidative stress markers and cardiovascular parameters, our results showed a significant increase on the TC, TG, HDL, LDL, CK, LDH, GLC, SBP, DBP immediately after exercise. Likewise, TC and LDL as well as the MDA, CK and LDH demonstrate a significant decrease after supplementation of *Opuntia ficus‐indica* juice at P1 and P2. Nevertheless, no significant effect on HDL, GLC levels or the DBP was observed after supplementation on *Opuntia ficus‐indica* juice.

Several studies showed that *Opuntia ficus‐indica* juice has a more important antioxidant capacity than other fruits (Seeram et al., 2008; Yahia & Mondragon‐Jacobo, 2011). These findings support our pick of the variety studied. Cactus pear contains compounds with antiradical action (i.e., phenolics, flavonoids and pigment compounds) (Maataoui et al., 2006; Tesoriere et al., 2008). These compounds were found to possess a high potential for capturing free radicals. Consistent with previous studies, the evaluation of the antioxidant activity indicate that the *Opuntia ficus‐indica* juice studied contain substances which are able to inhibit the action of free radicals as DPPH^●^ (Maataoui et al., 2006; Tesoriere et al., 2008). The choice of the DPPH^●^ method is in accordance with that of Fernandez‐Lopez et al. (Bloomer & Cole, 2009).

It is well accepted that the physical exercise is characterized by an increment in the volume of oxygen consumed (Powers & Jackson, 2008) which leads to an increase in ROS production and causes oxidative stress and muscle damage (Hammouda et al., 2012). As a result, an elevation of LDH and CK levels will be observed after prolonged exercise. Likewise, Baird, Graham, Baker, & Bickerstaff (2012) and Ceci et al. (2014) showed that acute exercise provokes micro‐lesions in the active muscle. This damage causes an increase in plasma concentrations of CK and LDH which leads to the decrease of the performance (Brancaccio, Lippi, & Maffulli, 2010). Indeed, the increase of CK rate indicates that the athlete is more vulnerable to injury and muscle damage caused by an intense physical activity. Our results illustrate that CK levels are significantly higher P2 above P1, before supplementation for EG as well as CG. Similarly, our findings showed that plasma concentrations of LDH recorded on P2 are significantly higher than those at P1 in EG and CG. Indeed, the study of Bloomer & Cole (2009) clearly supports an increase in the CK and LDH concentrations in the blood in response to intense exercise.

Concerning the effect of exercise on lipid peroxidation, our data revealed a significant increase in MDA levels during the YYIRT. Our data agree with those of Bouzid, Hammouda, Matran, Robin, & Fabre (2014) which demonstrate that there are two possibilities to explain the elevation of lipid in response to exercise. Foremost, this increase is due to a greater generation of ROS. Secondly, the degree of fatty acid desaturation affects lipid peroxidation level. In a same context, Baker, Bailey, Hullin, Young, & Davies (2004) noted an increase in both lipid hydro‐peroxides and MDA immediately after a sprint exercise. Regarding the effect of exercise on biochemical parameters and in accordance with previous reports (Antoncic‐Svetina et al., 2010; Hammouda et al., 2012), the present data showed a significant increase of these parameters (i.e. TC, TG, HDL‐cholesterol and LDL‐cholesterol, GLC) at P2 versus P1 for EG and even for CG. These findings are in‐line with those of Hammouda et al. (2013) which indicated that the intensity of YYIRT is sufficient to increase biochemical parameters. Furthermore, this increase in lipid profile reflects the mobilization of purine cycle following this type of exercise.

Regarding blood pressure, our results indicate that the values of DBP and SBP recorded following the YYIRT are significantly higher than those at rest for the EG as well as CG.

Concerning supplementation, *Opuntia ficus‐indica* juice leads to an improvement on YYIRT performance (i.e., *V*O_2max_, VMA). In concordance with previous research (Hammouda et al., 2013), it seems that the enhancement of the YYIRT performance presented in this study is essentially due to the decrease of muscle damage and the rating of perceived exertion (RPE). In addition, CK and LDH levels present a significant decrease which is observed after supplementation with *Opuntia ficus‐indica* juice in EG compared to CG at P2 in the current work. Our outcomes are consistent with Kato, Kurakane, Nishina, Park, & Chang (2013) which have shown that ingestion of Acanthopanax sieboldianus (antioxidant) before a high‐intensity exercise reduces LDH concentrations in the blood and decreases the sensation of fatigue caused by exercise.

Concerning heart rate, supplementation with *Opuntia ficus‐indica* juice causes a significant decrease on HRmax for the EG after. Nevertheless, no significant difference was observed in CG. These data coincide with those found by Schmitt, Fouillot, Nicolet, & Midol (2008) who recorded a decrease in HR (−9 beats/min) for elite athletes after taking an extract of *Opuntia ficus‐indica* (5 g/kg body weight). Likewise, the supplementation of *Opuntia ficus‐indica* juice has no effect on the DBP or in the SBP. In this context, few studies in the field have focused on the measurement of blood pressure after antioxidant supplementation (Champagne, 2006; Saneei, Salehi‐Abargouei, Esmaillzadeh, & Azadbakht, 2014). The studies found in the literature are controversial. In fact, in accordance with our results, Ravn‐Haren et al. (2013) showed no significant effect of 500 ml/day of *Apple* juice supplementation on blood pressure. However, Dohadwala & Vita (2009) have shown a decrease in blood pressure after the consumption of antioxidants. Likewise, Salonen, Slater, Tuomilehto, & Rauramaa (1988) have suggested that supplementation with *Opuntia ficus‐indica* can be used as an antihypertensive therapy. There is a possible explanation in the difference in both the kind of the antioxidant supplemented and the type of the exercise performed.

Moreover, our findings showed that supplementation with *Opuntia ficus‐indica* juice for 2 weeks can reduce oxidative stress of exercise with a significant decrease in MDA levels compared to before supplementation. Our results confirm those found in the literature (Deldicque et al., 2013; Van Proeyen, Ramaekers, Pischel, & Hespel, 2012) that supplementation of *Opuntia ficus‐indica* juice improves aerobic performance and reduces oxidative stress. Consistent with our data, Tesoriere et al. (2008) investigated the antioxidant activity of *Opuntia ficus‐indica* in 18 healthy volunteers (250 g of fresh fruit or 75 mg of vitamin C) twice daily for 2 weeks. This supplement decreases the oxidative damage and improves the antioxidant status. In this context, the study of Jówko et al. (2011) found that dietary with antioxidant supplementation (green tea extract) could enhance antioxidant status and reduce tissue damage from oxidative stress occurred during the exercise. The production of free radicals in response to physical exercise is an important source of muscle damage. Thus, to protect themselves from oxidative stress and maintaining the redox balance in the most difficult periods of a sports season in mixed disciplines (aerobic, anaerobic), the athlete must increase the nutritional content of antioxidants to neutralize free radicals (Jówko et al., 2011; Teixeira, Valente, Casal, Marques, & Moreira, 2009).

Concerning HDL‐cholesterol concentrations, our results revealed a significant increase after YYIRT in both EG and CG. Similarly, the TC and TG levels after supplementation is significantly lower than before supplementation in the EG. However, no significant difference was observed in CG. According to Wolfram, Kritz, Efthimiou, Stomatopoulos, & Sinzinger (2002), consumption of a variety of the *Opuntia ficus‐indica* for 8 weeks causes a decrease in TC (12%), LDL cholesterol (15%), the apolipoprotein B (9%), TG (12%), GLC (11%), and insulin (11%). Another study showed that *Opuntia ficus‐indica* reduced cholesterol levels in human blood and altered the composition of LDL (Stintzing & Carle, 2005).

For GLC values, our data indicated that the two groups behaved in the same manner. Indeed, these concentrations increased significantly with YYIRT for the EG and even for CG before as well as after supplementation. Cicero, Derosa, & Gaddi (2004) showed that the *Opuntia ficus‐indica* supplements can also be effective in reducing GLC levels. In the same way, the work of Feugang et al. (2006), showed that *Opuntia ficus‐indica* contain fiber and pectin, which can reduce the level of GLC. Likewise, Trejo‐González et al. (1996) demonstrated the hypoglycemic activity of the extracts of *Opuntia ficus‐indica* on diabetic individuals.

### 4.1 Limits of study

This study has some obvious limitations. The first concerns samples taken. It will be better that we make a belated sample to know the impact of *Opuntia ficus‐indica* juice supplementation on recovery. In addition, the ideal would be the addition of another group; a placebo group or group who receives another antioxidant supplementation. One more limitation is that, in the present work, we did not carry away the effect of *Opuntia ficus‐indica* supplementation in the recovery period. No women were available for the study. Another limitation about methods to access antioxidant activity (FRAP, ABTS). Future studies should highlight other markers of oxidative stress (i.e., carbonated protein, glutathione, total antioxidant status etc.).

## 5 CONCLUSION

In conclusion, we conclude that 14 days of supplementation with *Opuntia ficus‐indica* juice has a potent antioxidant activity makes it possible to reduce total and LDL‐cholesterol with only a moderate lowering of HDL‐cholesterol and oxidative stress. Moreover, supplementation decreases muscle damage caused by the endurance exercise.

## CONFLICT OF INTEREST

The authors declare that they have no competing interests.
